# Noise reduction and mask removal neural network for X-ray single-particle imaging

**DOI:** 10.1107/S1600576721012371

**Published:** 2022-02-01

**Authors:** Alfredo Bellisario, Filipe R. N. C. Maia, Tomas Ekeberg

**Affiliations:** a Laboratory of Molecular Biophysics, Department of Cell and Molecular Biology, Uppsala University, Husargatan 3 (Box 596), SE-751 24 Uppsala, Sweden

**Keywords:** coherent X-ray diffractive imaging (CXDI), free-electron lasers, diffract-then-destroy, protein structures, single particles, XFELs, imaging

## Abstract

Free-electron lasers could enable X-ray imaging of single biological macromolecules and the study of protein dynamics, paving the way for a powerful new imaging tool in structural biology, but a low signal-to-noise ratio and missing regions in the detectors, colloquially termed ‘masks’, affect data collection and hamper real-time evaluation of experimental data. In this article, the challenges posed by noise and masks are tackled by introducing a neural network pipeline that aims to restore diffraction intensities. For training and testing of the model, a data set of diffraction patterns was simulated from 10 900 different proteins with molecular weights within the range of 10–100 kDa and collected at a photon energy of 8 keV. The method is compared with a simple low-pass filtering algorithm based on autocorrelation constraints. The results show an improvement in the mean-squared error of roughly two orders of magnitude in the presence of masks compared with the noisy data. The algorithm was also tested at increasing mask width, leading to the conclusion that demasking can achieve good results when the mask is smaller than half of the central speckle of the pattern. The results highlight the competitiveness of this model for data processing and the feasibility of restoring diffraction intensities from unknown structures in real time using deep learning methods. Finally, an example is shown of this preprocessing making orientation recovery more reliable, especially for data sets containing very few patterns, using the expansion–maximization–compression algorithm.

## Abstract 2

A neural network for coherent X-ray diffractive imaging experiments is presented that can restore noisy and masked simulated diffraction intensities from biological macromolecules.

## 1. Introduction

Knowing the structure of biological macromolecules, such as proteins, is fundamental to understanding their mechanisms and function. Historically, X-ray crystallography has been the most successful tool for structure determination, and it is still the most commonly used technique. The major drawback is that the proteins must be crystallized, which is not always feasible and makes it hard to study protein dynamics. Single-particle imaging (SPI) methods aim to investigate biological structures without the need for crystallization. One SPI method that has shown impressive results is electron microscopy (EM). Cryogenic EM (cryo-EM), in particular, is routinely used to study protein structures and dynamics. Yet cryo-EM is limited in time resolution, as it cannot reach time scales much faster than milliseconds (Chen & Frank, 2015 ▸).

X-ray free-electron lasers (XFELs) are the latest generation of accelerator-based light sources and can produce coherent X-ray pulses shorter than 100 fs. XFELs have enabled the use of X-rays for SPI experiments. The underlying principle behind X-ray SPI experiments is known as ‘diffraction before destruction’ (Chapman *et al.*, 2006 ▸). In such experiments, the X-ray pulse hits a stream of biological samples injected into a vacuum chamber. The pulse damages the sample, and the entire structure is fragmented into ions on the time scale of a few femtoseconds (Hau-Riege *et al.*, 2004 ▸; Jurek *et al.*, 2004 ▸; Bergh *et al.*, 2008 ▸). The femtosecond duration of the X-ray pulse guarantees that the scattered photons carry information about the structure before the effects of radiation damage take place (Neutze *et al.*, 2000 ▸). Since the first demonstration of this method, XFELs have been used to determine the structures of several types of samples, such as cells (Mancuso *et al.*, 2010 ▸; Seibert *et al.*, 2010 ▸; van der Schot *et al.*, 2015 ▸), cell organelles (Hantke *et al.*, 2014 ▸) and viruses (Seibert *et al.*, 2011 ▸; Kassemeyer *et al.*, 2012 ▸; Daurer *et al.*, 2017 ▸), and even to resolve the three-dimensional structure of viruses (Ekeberg *et al.*, 2015 ▸).

Achieving atomic resolution for small biological structures, such as proteins, is challenging because it requires a low background and a high hit rate. Photons scattered from the beamline’s injection gas and optical instruments will create a background signal that is often comparable in strength to the signal originating from the sample. Furthermore, the data will be incomplete because of ‘missing’ regions, where no diffraction is collected due to, for example, a beam stop or gaps between individual detector modules.

Additionally, photon detectors can only measure the intensity of the scattered beam but not the phase. Phase-retrieval algorithms allow one to determine phases from an oversampled signal (Fienup, 1978 ▸), yet solving such a problem requires computational effort and experimental data with a sufficiently strong signal. Depending on the sample size and the desired resolution, the number of photons needed varies, and it is necessary to take into account noise (Martin *et al.*, 2012 ▸) and missing data to ensure the convergence of the algorithms.

Applications of neural networks have been blooming in the past decade, and the technology can now often be used without requiring an unreasonable computational effort or huge data sets thanks to ‘transfer’ learning methods and new hardware technology. Machine learning techniques are interesting for future experiments at XFEL facilities which are undergoing a rapid increase in their data collection rate. X-ray detectors can generate up to 15 Gbyte s^−1^ of raw data (Muennich *et al.*, 2016 ▸) and machine learning solutions may be helpful to improve and speed up data analysis. Up to now, only a few studies have addressed this potential directly for X-ray SPI experiments, employing neural networks for image classification (Langbehn *et al.*, 2018 ▸; Shi *et al.*, 2019 ▸; Zimmermann *et al.*, 2019 ▸; Ignatenko *et al.*, 2021 ▸), for defect identification and phase retrieval (Cherukara *et al.*, 2018 ▸; Lim *et al.*, 2021 ▸; Wu, Juhas *et al.*, 2021 ▸; Wu, Yoo *et al.*, 2021 ▸), for shape and orientation recovery of silver nanoclusters (Stielow *et al.*, 2020 ▸), and for the reconstruction of electron densities of metallic nanoparticles from experimental data of the 3D Fourier space (Chan *et al.*, 2020 ▸).

In an X-ray SPI experiment, each diffraction pattern is a noisy and incomplete sampling of unknown Fourier magnitudes in a 3D volume at an unknown orientation. In this paper, we investigate deep learning as a tool to denoise and demask diffraction patterns. The goal is to provide a tool that can restore diffraction intensities from unknown protein structures to support future online analysis, for example in hit finding, diffraction pattern classification and other experimental diagnostics. Moreover, restoring diffraction patterns allows us quickly to calculate autocorrelation functions without the severe artefacts caused by missing data.

## 2. Method

### 2.1. Data set simulation

To perform denoising and demasking under realistic experimental constraints, we need a tool that can handle the diffraction signal from any structure at any orientation, since during experiments no previous knowledge is assumed about the sample or its orientation. Our method achieves this by training a neural network with simulated diffraction patterns from proteins chosen at random from the Protein Data Bank (PDB) (Berman *et al.*, 2000 ▸).

We downloaded 10 900 atomic coordinates files, each randomly selected, without repetitions, from proteins within the molecular weight range of 10–100 kDa. From each file, only one diffraction pattern was then simulated. During SPI imaging, the sample weight is usually known and the selection does not represent a significant limitation to the generality of the algorithm. Diffraction patterns were simulated using the *Condor* package (Hantke *et al.*, 2016 ▸), setting the photon energy to 8 keV. The detector was assumed to be placed 15 cm from the interaction region and the pixel size was 200 µm.

**Figure 1:**
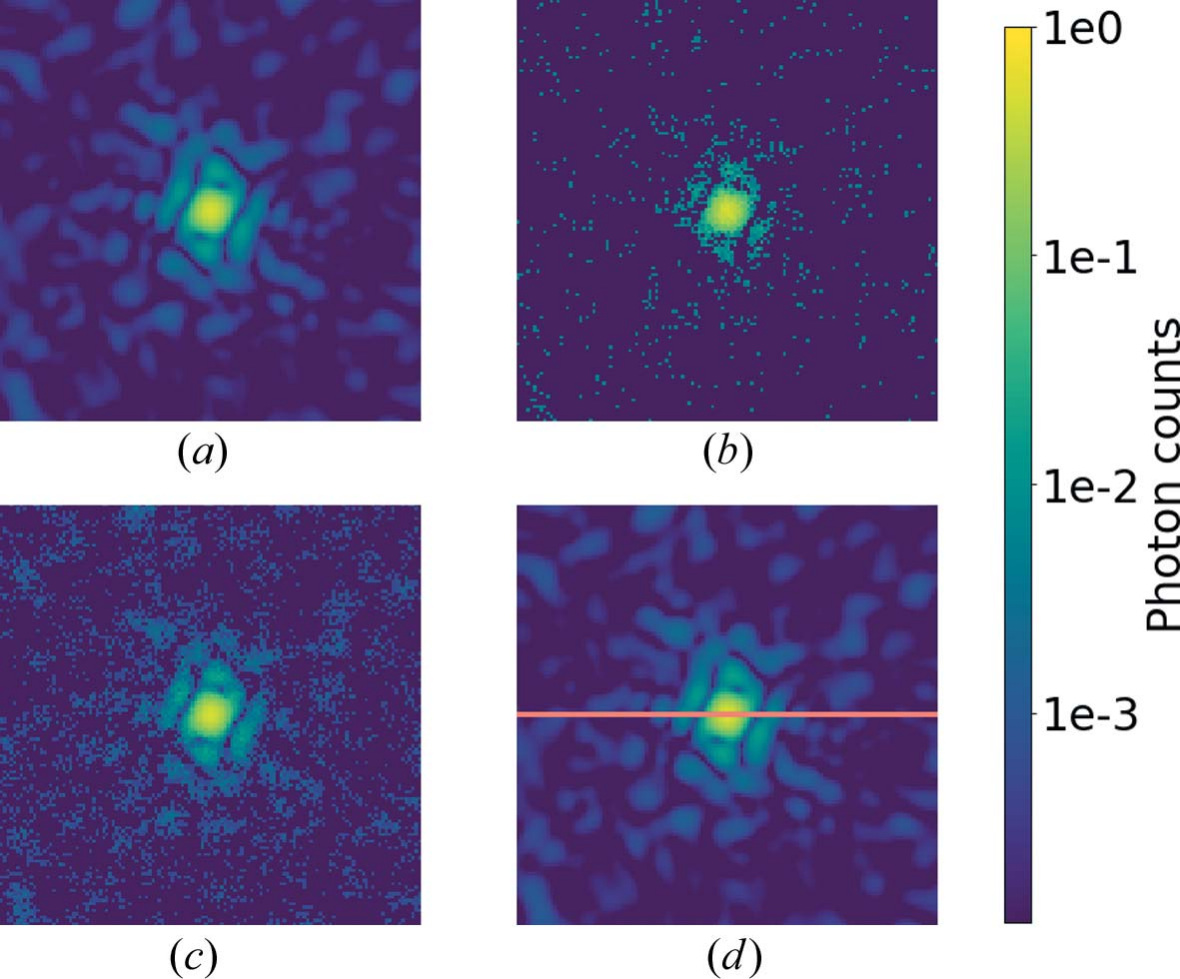
Simulated diffraction patterns from a *Burkholderia cepacia* lipase (PDB 3lip; Schrag *et al.*, 1997 ▸), logarithmic scale colour map. (*a*) A simulated diffraction pattern without noise. (*b*), (*c*) Poisson-sampled diffraction patterns. (*d*) A masked simulated diffraction pattern without noise. Orange pixels represent missing data.

Several sources of noise could affect a diffraction experiment, but in our study we limited ourselves to Poisson noise, which is by far the most dominant. First, we normalized all the noiseless patterns to set them to the same maximum intensity. We then downsampled each pattern to 128 × 128 pixels to reduce the computational effort and rescaled with a number we will refer to as the intensity factor before applying Poisson noise. This gave us control of precisely how strong the signal is in each pattern. Fig. 1 ▸ shows an example from our simulated data set.

### 2.2. Data preprocessing

Neural networks often struggle when the dynamic range of the input is high. For this reason, we applied a log-scale normalization to keep values within the range [0, 1] and further reduce the dynamic range. Log-scale normalization was performed by applying the following conversion, 

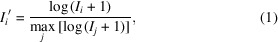

where *I*_*i*_ is the value of the pattern at pixel *i* and 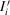 is the corresponding normalized value.

To understand how the size of the missing regions affects the recovery of the signal, we studied a mask composed of a stripe across the entire detector and tested our model for mask widths of increasing size. We also tested the network on a mask corresponding to the missing regions on the AGIPD photon detector currently used in Hamburg at the European XFEL facility.

### 2.3. Neural network pipeline

We implemented our pipeline using *Keras* (Chollet *et al.*, 2015 ▸) and *Tensorflow* (Abadi *et al.*, 2015 ▸). The deep learning pipeline was designed following a model architecture known as U-Net (Ronneberger *et al.*, 2015 ▸), originally developed for image segmentation in biomedical sciences. U-Net is a convolutional neural network inspired by autoencoders and residual networks.

Convolutional autoencoders are networks developed to learn the representation of a data set efficiently and to perform dimensionality reduction. The first part of an autoencoder is called the encoder, which compresses the input into a set of feature maps that are then upsampled and decoded by the second part of the network, the decoder. The Fourier transform of a single particle can be sampled more finely than the Nyquist–Shannon rate, allowing compression without any loss of information. The same is true even for noiseless diffraction patterns without phases, but the compressed information content will be the size of the autocorrelation instead of the size of the sample. For this reason, autoencoders seem especially well suited for denoising diffraction data.

**Figure 2:**
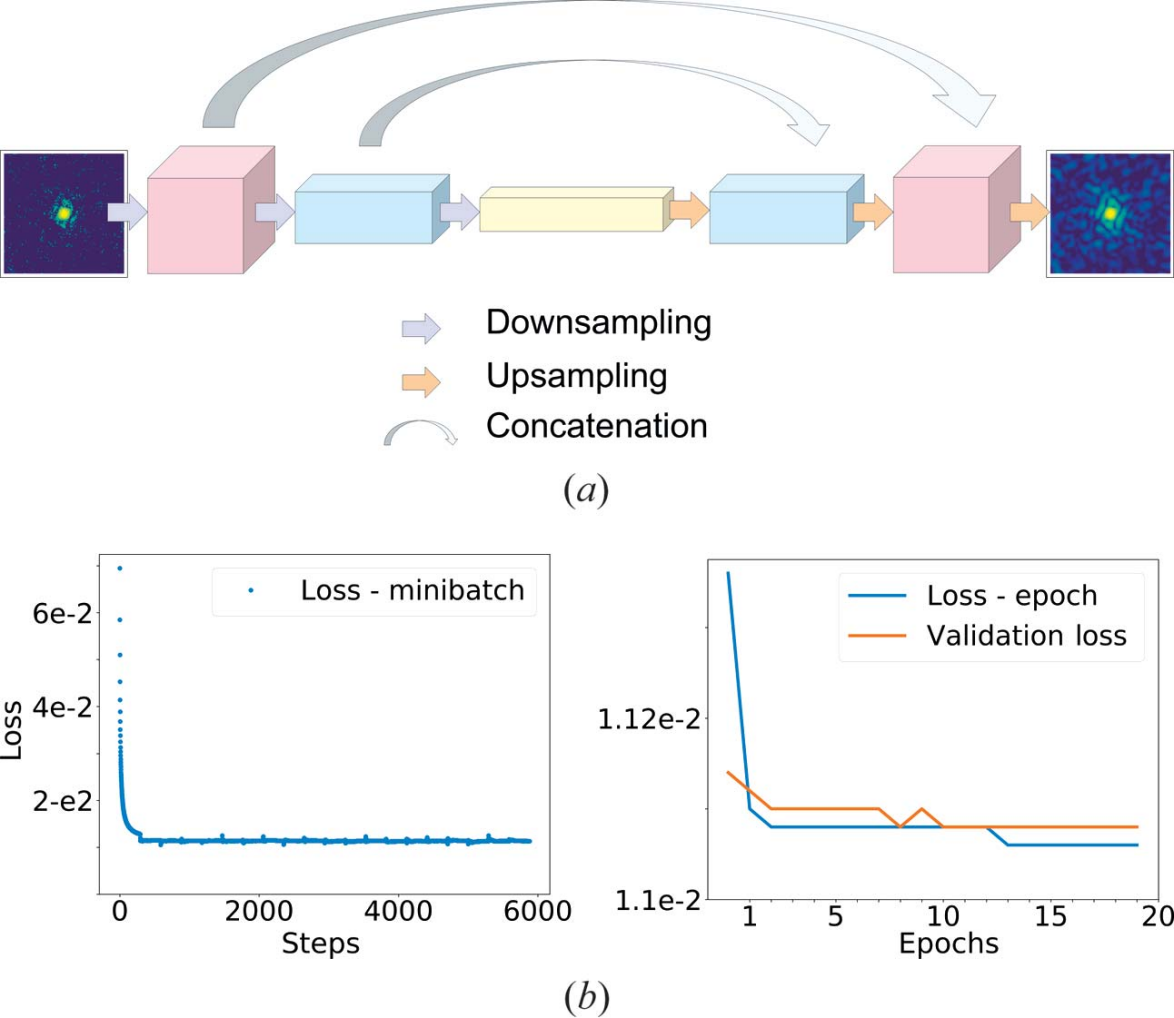
(*a*) The U-Net schematic structure. Concatenations between symmetrical layers are the key difference of U-Nets over standard convolutional neural networks and autoencoders. In our model, we also applied dropout layers in the encoder for regularization. Dropout layers are used for further implicit data augmentation. (*b*) Examples of loss functions obtained during training for denoising at an intensity factor of 10. Minibatch loss quickly drops and oscillates within the range of the validation loss. Each sudden change in the minibatch loss function after 294 steps (one epoch) is due to the effect of dropout layers. The minibatch loss function is reported for better visualization of the loss decay. Validation decays slowly and mostly overlaps the training loss.

U-Net supplements the autoencoder network with skip-layers (Fig. 2 ▸), providing the decoder with features from the encoder that can now bypass the bottleneck. This helps the convergence during network training. In addition, dropout layers are used to reduce the overfitting effect and applied after each of the last two downsampling steps. During each training epoch, these layers randomly disable 50% of the following hidden layers, setting weights to zero and forcing other units in the layer to be activated.

The U-Net is trained using an *Adam* optimizer (Kingma & Ba, 2014 ▸) to minimize a binary cross-entropy loss function between the output and the noiseless pattern. We divided the data set into three ensembles. The first ensemble contains 9900 images, 5% of which were used as a validation data set while the rest were used for training. Another 1000 images were used to test the trained network. There is no overlap between the training, test and validation data sets. Each image in the data sets corresponds to a different protein in a random orientation. For each task, noise level and mask, we retrained the network from scratch for 20 epochs. Each training phase took around 30–40 min running on an RTX 2080 Ti GPU card. Details regarding the implemented model, a portion of each data set and the weights of the neural network are deposited within a dedicated repository (Bellisario *et al.*, 2021 ▸).

### 2.4. Binary filter

The inverse Fourier transform of the diffraction intensities of a protein represents the spatial autocorrelation function of its electron densities in real space. If the autocorrelation support is known, it is possible to constrain the auto­correlation space by applying a binary filter, which sets every pixel that does not belong to the support to zero. By applying an inverse Fourier transform to the filtered autocorrelation function, we can retrieve denoised diffraction intensities. This is equivalent to applying a low-pass filter to reduce the local variance of the diffraction intensities. In our case, we could apply an almost optimal binary filter for each diffraction pattern, as we could derive the true autocorrelation from the noiseless simulated intensities. We applied this simple denoising strategy to our noisy test data set to benchmark the results presented in this paper.

More elaborate algorithms have been introduced to solve phase retrieval while also restoring intensities for sparse and missing data (Pietrini & Nettelblad, 2018 ▸), achieving remarkable results. However, these algorithms require a long computation time, making them less suitable for real-time evaluation.

### 2.5. Orientation recovery

Orientation recovery over denoised and demasked intensities was performed using a modified version of the expansion–maximization–compression (EMC) algorithm (Loh & Elser, 2009 ▸) that can work with floating numbers. In particular, while standard EMC uses a Poisson distribution for the measured photons, we assumed a Gaussian distribution to work with continuous and normalized data from U-Net. We benchmarked EMC on U-Net outputs against a variation of EMC designed for data-starved problems. For a very few patterns, the oriented intensities will not cover the entire Fourier space. To overcome this, we convolve the 3D Fourier model after each iteration with a 3D Gaussian kernel with a standard deviation smaller than the Shannon pixel size. This will distribute each pixel value in the pattern over many voxels in the 3D model, thus increasing the overlap between patterns. We will refer to this approach hereinafter as blurred EMC.

## 3. Results and discussion

### 3.1. Noise reduction

**Figure 3:**
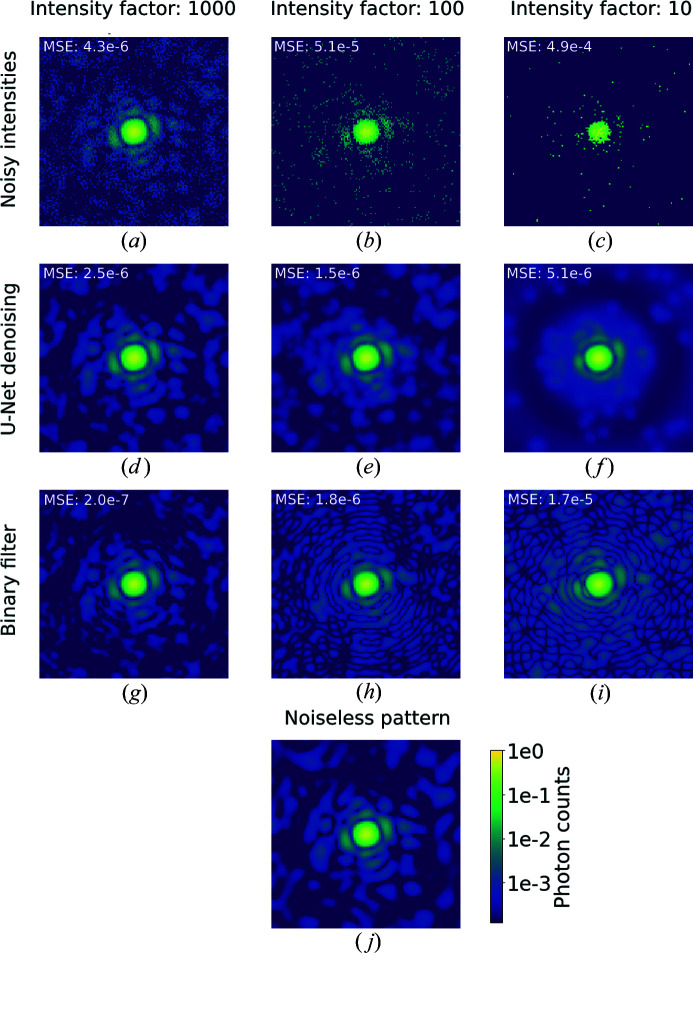
Diffraction patterns from *Fusarium oxysporum* trypsin (PDB 1fn8; Rypniewski *et al.*, 2001 ▸), logarithmic scale colour map. (*a*)–(*c*) Poisson-sampled diffraction patterns at intensity factors of 1000, 100 and 10, respectively. (*d*)–(*f*) Denoised output from U-Net. (*g*)–(*i*) Denoised output from the binary filter. (*j*) A noiseless diffraction pattern. The mean squared errors (MSEs) of each reconstruction with respect to noiseless intensities are reported in the top left-hand corner of the patterns.

First, we trained U-Net to denoise and compared its results with the binary filter approach. Fig. 3 ▸ shows diffraction patterns from *Fusarium oxysporum* trypsin (PDB 1fn8) and the corresponding denoised intensities using both U-Net and autocorrelation denoising. These, and all the denoised images shown in this paper, come from the respective test data sets and the neural network did not have access to them during training.

Both U-Net and autocorrelation filtering produce visually convincing results. Both suffer from low signals at high scattering angles but produce a dramatic improvement even for the noisiest case. To quantify the performance of these pipelines, we calculate the mean-squared error (MSE) metric between the result and the original noiseless patterns. For this comparison, the U-Net output has to be linearized by inverting the logarithmic normalization applied earlier. The MSE is a useful metric for these applications since it is preserved by the Fourier transform and thus also represents the error in the autocorrelation of the pattern.

**Figure 4:**
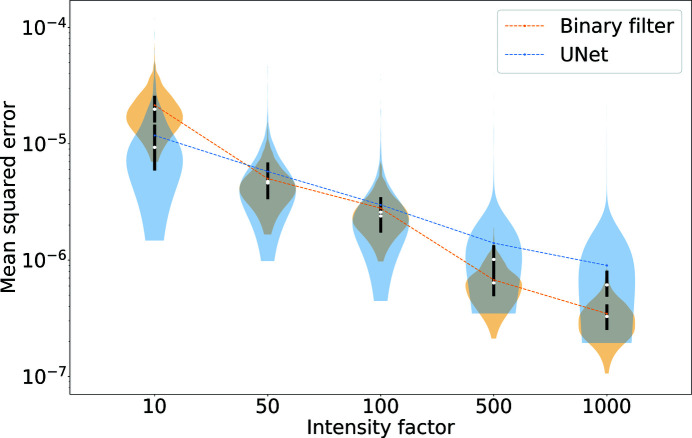
A violin plot for the MSE distributions at different noise levels. Each violin reports the full distribution of the MSE values within the test data set at the respective intensity factor. The gap in the bar represents the median value, while the black area corresponds to the interquartile ranges 25–75%. Dashed lines connect the mean values for each distribution.

Fig. 4 ▸ shows the MSE distributions as a function of the intensity factor. The denoising task becomes more challenging for both methods as the signal decreases, and the autocorrelation filter outperforms U-Net somewhat when the signal is strong, while for low signals the relation is reversed. While we are especially interested in low-signal conditions, the good performance of the binary filter implies that its application has to be favoured for high diffraction signals. Yet binary filters are rarely used in practice as they are notoriously bad at handling missing data, as we will discuss in Section 3.3. We also have to consider that the values for the binary filter represent a best-case scenario by using the already known autocorrelation. In contrast, our neural network did not require knowledge about the noiseless intensities to obtain the reported results. U-Net is a parameter-free model, and in this sense it could be a quicker and more reliable approach for online analysis. Furthermore, linearization negatively impacts the performance of U-Net, as, by downsampling to smaller images, the local maxima are averaged, and we need to fit the noisy patterns to find their value. We also note that U-Net introduces fewer visual artefacts even when the MSE is slightly worse.

The variance of the MSE distributions is higher when denoising the test data set with U-Net, as shown in Fig. 4 ▸. The outliers in the MSE distributions correspond to large proteins with a smaller speckle size, or diffraction patterns with strong anisotropy. This problem seems to be related to the data set as these images are not well represented during training. We conjecture that data augmentation and a larger training data set could remove outliers without overfitting the model.

In many computer vision applications the performance of image processing models is studied in terms of peak signal-to-noise ratio (PSNR). In our case the PSNR can be directly derived from the MSE as

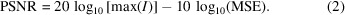

Table 1 ▸ reports the average PSNR for reconstructions obtained with both of the denoising algorithms and for noisy inputs.

We further tested how robust the network is to ensemble bias, that is, how well it performs on diffraction data that are not drawn from the same ensemble as the training data. We decided to consider two scenarios. First, we tested its robustness to non-protein-like structures by considering small geometric objects. Second, we simulated data from larger biological structures that should correspond to the current state-of-the-art capabilities of X-ray SPI.

To achieve this, we simulated additional diffraction intensities from a cube, a sphere and an icosahedron of size 4 nm and the same average density as a protein (1.35 g cm^−3^) (Fig. S1 in the supporting information). These objects produce diffraction patterns with decidedly different speckle structures and a strong intensity drop at high scattering angles. Current X-ray SPI experiments provide a resolution comparable to the typical size of a 10 kDa protein, so for the second scenario we decided to test our network on virus-like nanoparticles. We simulated one pattern per new sample, choosing the biological assembly data of BDV T1 virus-like nanoparticle (PDB 1wcd) (Coulibaly *et al.*, 2005 ▸), Seneca Valley virus (PDB 3cji) (Zhang *et al.*, 2003 ▸) and Dengue virus capsid (PDB 1p58) (Venkataraman *et al.*, 2008 ▸), with respective diameters of 25, 40 and 60 nm. To preserve the oversampling, we moved the detector further back for these simulations, just as one would for a normal experiment on samples of this size. We set the detector distance to 2.67 m for every particle to compare them. We also simulated PDB 1wcd at 2 m detector distance to optimize the central speckle width within the range considered during model training.

U-Net manages to handle intensities from these new simulations with an MSE within the expected range, as shown in Table 1 ▸. Its solid performance on these patterns suggests that U-Net has indeed learned features relevant to denoising diffraction intensities from unknown structures. We note that the BDV T1 virus-like nanoparticle has a relatively higher error than the others at the same detector distance. This is expected since the simulated central speckle was quite large and outside the training data set range. It also explains why a shorter detector distance significantly improved the MSE. As we can see from the MSE values in Table 2 ▸, our pipeline is in principle able to handle data from particles larger than proteins as long as the speckle size does not differ too much from the range covered by the training data set.

We extracted the true phases of the diffraction patterns from our *Condor* simulations and used them to derive the best possible 2D electron-density reconstructions given each set of noisy intensities. Assuming knowledge over phases is a strong assumption, but it is still useful to evaluate if denoising has an impact on the image’s resolution. This test is preferable to running a full phase retrieval since these results often vary strongly depending on the exact parameter choices. When using the square root of the intensities denoised with U-Net as amplitudes together with the correct phases, we noticed an improvement in the real-space image. For example, we could not distinguish between an icosahedron and a sphere in real space (Fig. S2) when using the lowest signals without denoising. On the other hand, the diffraction intensities of the cube were the most difficult to reconstruct, having the highest MSE. In real space, this manifests itself as fuzzy edges and a slight halo around the corners. Finally, we note that the cube, having long sharp edges, is the object here that is the most dissimilar to a protein and probably marks the boundary for what objects can still be handled by our U-Net.

**Figure 5:**
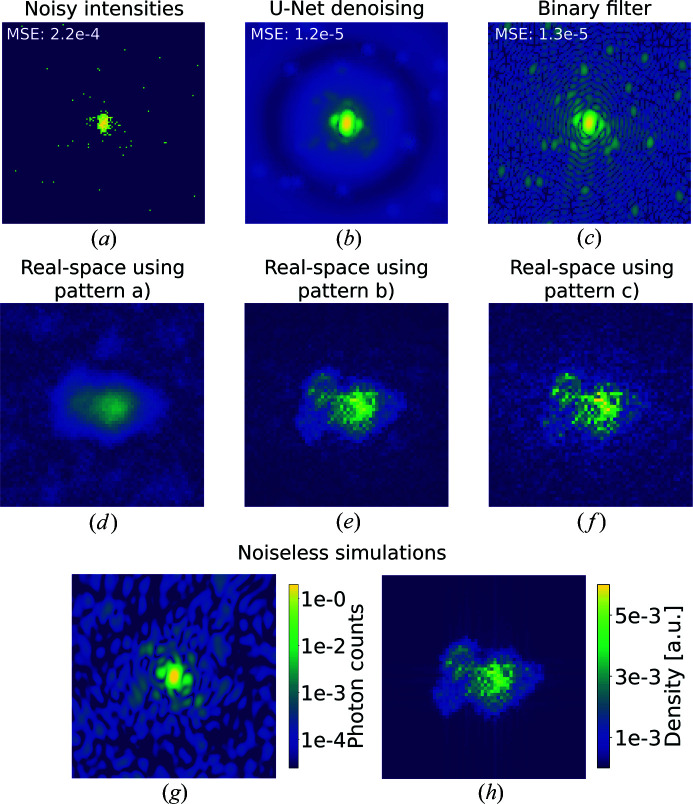
Diffraction intensities and real-space 2D electron densities of shark IgNAR (PDB 4hgm; Kovalenko *et al.*, 2013 ▸). The real-space image was derived using true phases and cropped for visualizing details. (*a*) Poisson-sampled diffraction intensities. (*b*) Denoised output from U-Net. (*c*) Denoised output from the binary filter. (*d*)–(*f*) Reconstructions obtained from, respectively, noisy intensities, U-Net output and binary filtered intensities. (*g*) A noiseless diffraction pattern. (*h*) The original structure.

Finally, we report the ‘best-case’ reconstruction performed for the 2D electron density of a protein from simulated phases. We compare the results obtained using amplitudes derived from the denoised intensities with the results from amplitudes derived from the output of the binary filter. As in the case above for the virus particles, the real-space structures in Fig. 5 ▸ were obtained from known simulated phases, thus representing the best possible outcome for the given amplitudes. We can observe how denoised amplitudes achieve higher-quality images than noisy ones, but the binary filtered intensities introduce more artefacts both around and within the protein support. This effect is probably caused by artefacts introduced in the pattern at high momentum transfer *q*. Nevertheless, denoised intensities with both methods seem to improve the image resolution, and binary filtered intensities lead to slightly noisier real-space images. As for the geometric shapes, the original noisy intensities introduce a blurring effect on the reconstruction. However, it is important to remark that this comparison does not prove that intensities denoised with U-Net will always improve the resolution or that phase retrieval would benefit from this process. Therefore, future work should be dedicated to investigating the impact of noise reduction with neural networks on phase retrieval.

### 3.2. Mask inpainting

**Figure 6:**
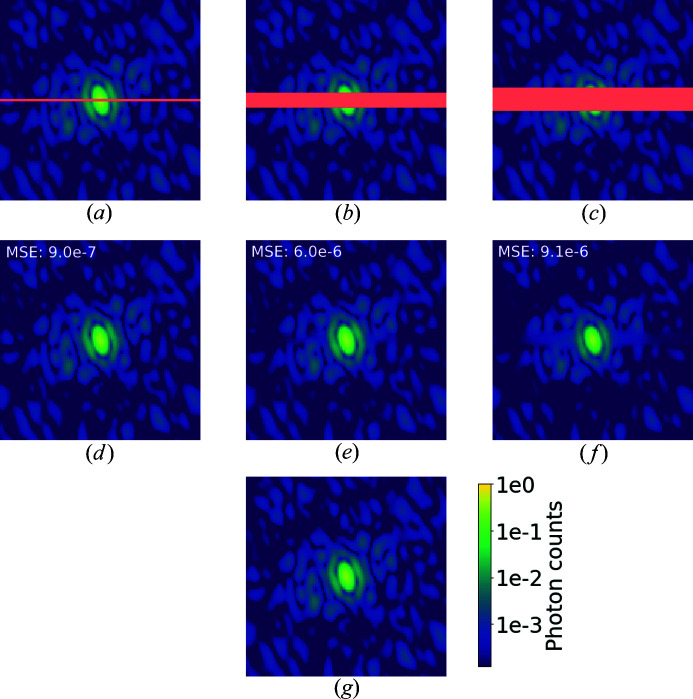
Diffraction patterns for human aurora A catalytic domain (PDB 4zs0; Kilchmann *et al.*, 2016 ▸), logarithmic scale colour map. Orange is used in the top row to highlight masked pixels. (*a*) A simulated diffraction pattern with a 2 pixel-wide mask. (*b*) A simulated diffraction pattern with a 10 pixel-wide mask. (*c*) A simulated diffraction pattern with a 15 pixel-wide mask. (*d*)–(*f*) U-Net demasked output. (*g*) Simulated diffraction intensities without a mask.

In this section, we consider masked but noiseless patterns as we aim to understand the ability of U-Net to fill in missing data. We tested the U-Net for a mask width of 2–20 pixels along the central row of the simulated intensities. In Fig. 6 ▸ we show an example of simulated diffraction intensities where differently sized missing regions have been inpainted using U-Net.

For small masks, the reconstruction is impressive and even high spatial frequencies are recovered. However, as the mask size increases, only low spatial frequencies are correctly recovered, while high spatial frequencies are recovered as an estimated local average. When the central speckle is entirely missing, the reconstruction becomes very difficult and not even the estimated central speckle resembles the ground truth.

**Figure 7:**
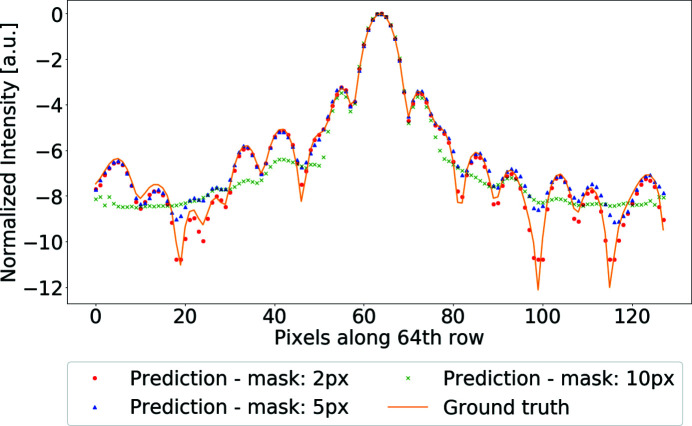
A profile of the recovered intensities along the central row for PDB 4zs0, plotted for three different mask sizes. When the mask is only a few pixels wide, all of the spatial frequencies are correctly retrieved, but the local minima are slightly overestimated. The central speckle size is roughly 22 pixels for this protein.

**Figure 8:**
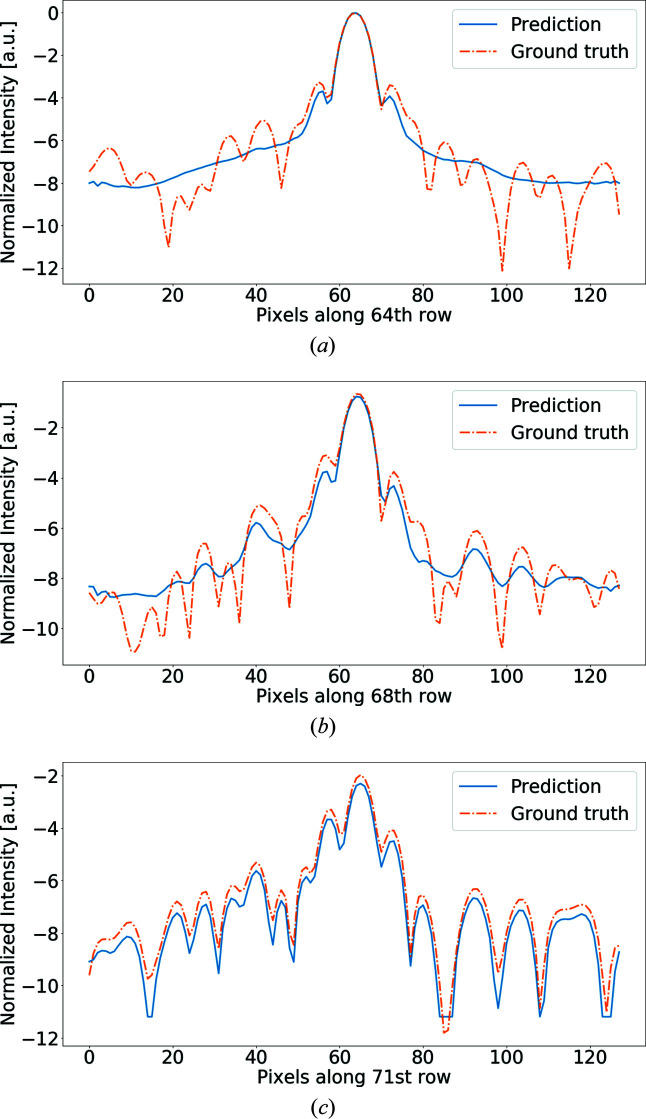
Profiles of the recovered intensities along three different rows for PDB 4zs0 and a mask size of 15 pixels. As we get closer to the unmasked data, we observe that the quality of the recovery improves significantly. In particular, the 71st row, which is the first row of masked data, is very accurate.

To give a better visualization of the differences between the recovered intensities and the true diffraction pattern, Fig. 7 ▸ shows the intensity profile for the central row of pixels in the mask. Even for small masks, local minima are usually slightly overestimated. This effect progressively increases for larger masks until intensities at high spatial frequencies are recovered as a local average and not as individual speckles. The distance from the unmasked regions seems to be significant for the reconstruction. In Fig. 8 ▸ we show three different rows from the same demasked pattern. The pixels for the 71st row are the closest to unmasked data. In this case, the prediction is very close to the shape of the simulated diffraction signal, while the intensities are only slightly underestimated. For rows further into the masked region, the profile gets closer to a local average of the expected intensities and gradually loses shape complexity.

**Figure 9:**
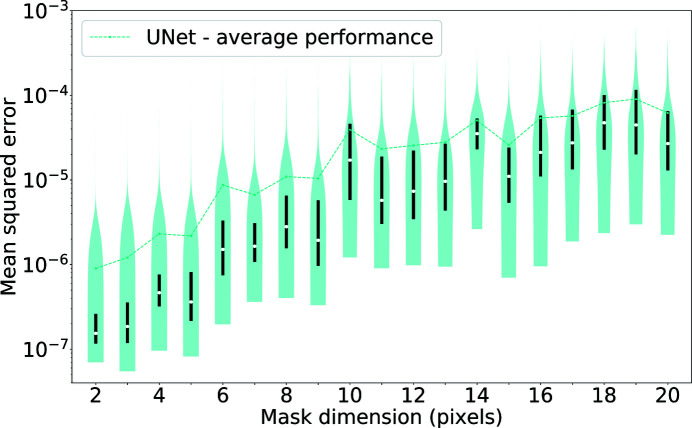
A violin plot of the MSEs calculated for increasing mask width. The average performance seems to depend linearly on the mask size for small masks. The trend for mean MSE distributions indicates that the dependence gradually saturates afterwards.

We report the MSE analysis for the neural network output in Fig. 9 ▸. Outliers beyond the third quartile for each mask class typically correspond to diffraction patterns with small central speckles compared with the mask, making the problem harder. In order to be able to compare these results with the denoising case, MSEs are calculated over the entire pattern and not only within the mask. The third quartile line always lies below 10^−5^ when the mask dimension is smaller than 10 pixels. This value corresponds to the average MSE when denoising in the noisiest scenario. After this point, the mask size is too wide to achieve good recoveries on the average pattern. Preliminary work on other mask shapes, such as circular beamstops (Figs. S3 and S4), showed a similar dependence on the diameter of the masked area.

**Figure 10:**
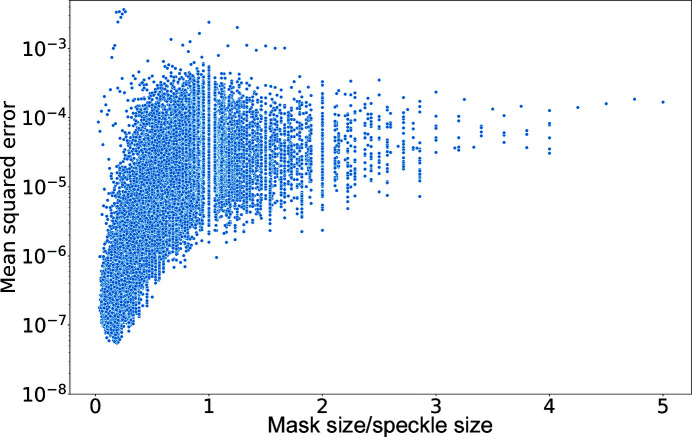
A scatter plot of the MSEs as a function of mask size over speckle size. Most of the patterns have a ratio of mask size to speckle size smaller than 1. Data are reported for all of the mask widths from Fig. 9 ▸.

In Fig. 9 ▸, the distributions have a high variance, especially for wide masks. Experience from phase retrieval shows that it is the ratio between the size of the missing region and the Shannon pixel that is relevant to how well we can reproduce the intensities in the masked area (Thibault *et al.*, 2006 ▸). To achieve a good recovery, experience shows that we require a mask size smaller than half of the central speckle. The scatter­plot in Fig. 10 ▸ reports MSE values for every mask as a function of the ratio between the mask width and the speckle size. For ratios smaller than 0.5, we can see a higher density of data with low errors, but at ratios closer to 1, the MSE values are higher and have a stronger variation. For even wider masks, the MSE values are consistently higher. The speckle size has been calculated as the distance between the two local minima of the central speckle along the vertical axis, the same as the shorter extent of the mask. Seventy-five per cent of the patterns in the entire data set have a central speckle that spans less than 25 pixels along the vertical axis, and the average central speckle size is close to 18 pixels. Most patterns have few data points for the low spatial frequencies when using mask widths of 10 pixels or greater. As the ratio between mask size and speckle size increases, the performance of the network clearly worsens.

### 3.3. Noise and mask

**Figure 11:**
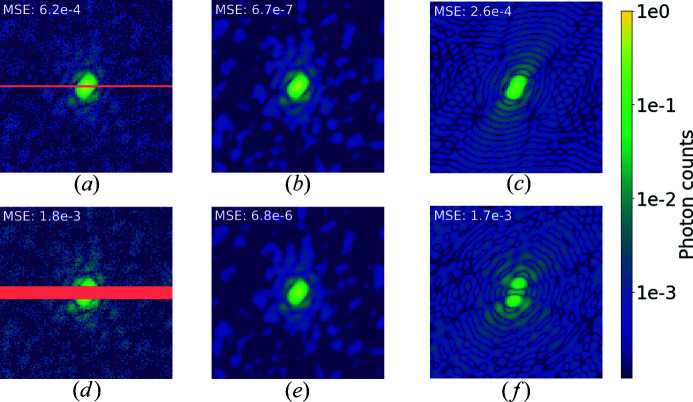
Diffraction patterns from leucyl-tRNA synthetase of *Candida albicans* (PDB 5agi; Zhao *et al.*, 2015 ▸) with two different mask sizes, logarithmic scale colour map. (*a*), (*d*) Masked and Poisson-sampled diffraction intensities. (*b*), (*e*) U-Net outputs. (*c*), (*f*) Results obtained using the binary filter constraint. As the mask size increases, the binary filter method becomes more and more ineffective, while the denoising capabilities of U-Net remain roughly unchanged.

Binary filters, which we compared with earlier in this article, can provide satisfactory results for denoising but are not robust to missing data regions. For this reason, this method is rarely applied for experimental data. However, as shown in the previous section, neural networks can inpaint masks, and it is reasonable to believe that they will perform much better than binary filters in the presence of both noise and mask. This section aims to investigate U-Net performance under this scenario to provide a proof of concept of their applicability for more realistic data. A qualitative comparison with binary filters is reported in Fig. 11 ▸.

**Figure 12:**
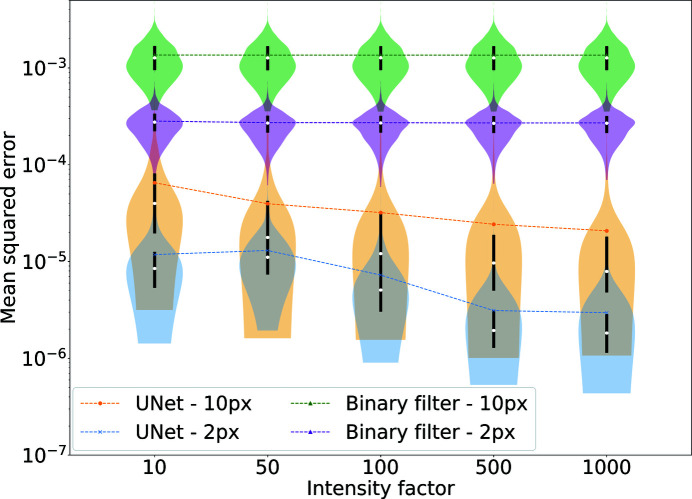
A violin plot for the MSE distributions. The MSEs are calculated between linearized denoised and demasked reconstructions and original intensities. There is a clear correlation between noise and MSE for the U-Net, while the performance of the binary filter is mostly affected by the size of the mask. Furthermore, a larger mask introduces a larger variance in the outputs due to patterns with speckle sizes close to or smaller than 10 pixels.

In Fig. 12 ▸ we report a comparison between these two methods considering different signal intensities and masks covering a width of 2 or 10 pixels along the central row. Comparing MSE distributions for the different methods at the same mask size and intensity factor, we can see that the average difference is larger than one order of magnitude and sometimes goes above two orders of magnitude. U-Net outperforms the binary filter under every condition and for every single pattern. U-Net was also robust to data from the geometric shapes used to test out-of-ensemble patterns, even when applying missing regions. We note that the mask dimension affects not only the MSE range but also the variance of the distribution. Given the results from the previous section, this might not be surprising. The smallest central speckle in our data set is only 7 pixels wide along the vertical axis, and the amount of missing information in a 10 pixel-wide mask is much larger than for the average pattern. The low effectiveness of the binary filter is almost independent of the intensity factor and only dependent on the mask size. Filtered intensities do not provide any improvement for data visualization unless the missing region is very small. In practice, the results from the autocorrelation filter would be even worse than presented here since we had access to optimal autocorrelations for filtering.

**Figure 13:**
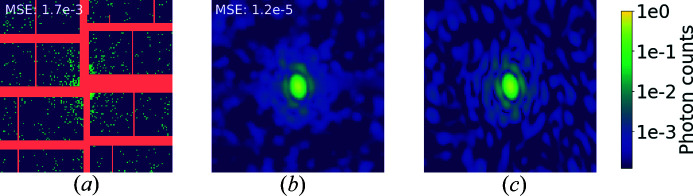
Diffraction patterns for an altered co-substrate of deacetoxy­cephalo­sporin C synthase (PDB 1hjg; Lee *et al.*, 2001 ▸), logarithmic scale colour map. (*a*) Poisson-sampled diffraction intensities with the mask from the AGIPD detector at the European XFEL. (*b*) U-Net denoised output with MSE. (*c*) A noiseless diffraction pattern without a mask.

Finally, we applied a realistic mask, taken from the AGIPD detector (Allahgholi *et al.*, 2015 ▸) employed at the European XFEL in Hamburg. An example is reported in Fig. 13 ▸. U-Net also manages to handle this irregular mask. As expected, intensities in large missing regions achieve a good estimate only for low spatial frequencies. MSE values for each reconstruction are within the expected range given the mask size, which is 14 pixels wide along the vertical axis for its widest area. The asymmetry in the AGIPD mask does not seem to affect the recovery of the intensities, and the reconstructions depend on the local width of the mask. The noise reduction is on a par with the performance in the unmasked case, which suggests that the denoising is mainly a local operation and does not suffer from large masks in other parts of the detector.

We then used our U-Net to restore simulated patterns of a single protein before attempting orientation recovery. We show how restoring missing data can help SPI data analysis, reducing the number of patterns required. We simulated diffraction at random orientations from phytochrome (PDB 4o01; Takala *et al.*, 2014 ▸) with around 1400 photons per masked pattern. We adjusted the experimental parameters to keep the Shannon pixel size within the range of the training data set. In particular, we simulated a photon energy of 6 keV, setting the detector distance at 3 cm from the interaction region and the pixel size to 200 µm. We applied a 2 pixel-wide mask positioned along the central rows. To reconstruct the 3D Fourier space model, we tested three EMC implementations: (i) regular EMC for noisy and masked patterns, (ii) floating-number EMC for U-Net outputs, and (iii) blurred EMC. We ran each version of EMC on data sets of different sizes ranging from 20 to 2000 images.

**Figure 14:**
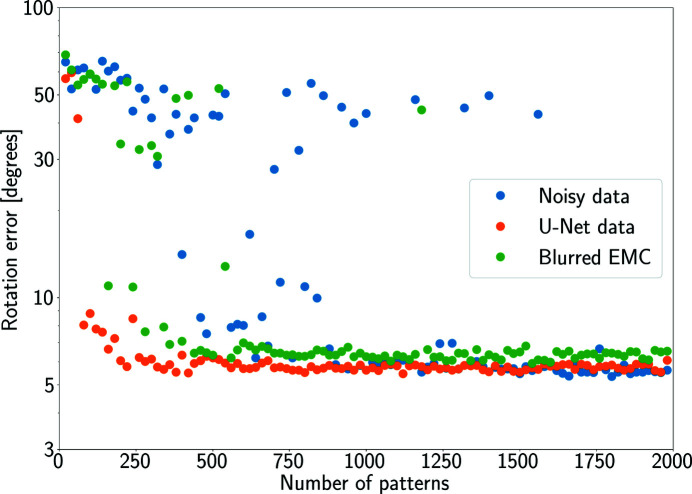
Average rotation error for the oriented patterns using EMC and blurred EMC. Blue markers refer to the EMC performance over masked and noisy intensities. Orange markers refer to the performance of EMC when using restored intensities. Blurred EMC (green markers) was applied to noisy and masked data only.

Fig. 14 ▸ shows that EMC requires around 700 noisy patterns to converge, while blurred EMC can run on fewer patterns but at the price of introducing higher errors on average. Even though EMC does not create better outcomes, given enough data the denoised and demasked data converged using significantly fewer patterns. Restored intensities also allow EMC to run more reliably with larger data sets.

## 4. Conclusions

In this work, we have provided a pipeline based on deep learning to denoise and demask diffraction images from X-ray single-particle imaging data. The neural network model has been trained and tested on simulated images, achieving satisfactory reconstructions. Our method has reduced high noise levels while also restoring data into missing regions better than binary filters. Demasking has been proved feasible within the reasonable limits imposed by lack of data at low spatial frequencies, and reconstructions of masked and noisy inputs have achieved satisfying results under realistic experimental settings. Specialized algorithms (Pietrini & Nettelblad, 2018 ▸) can be applied for interpolating missing data, per­forming denoising and demasking at the same time. However, these algorithms are not suitable for online analysis, as they require user evaluation and long computational time. Deep learning methods could become new and reliable tools for data visualization and preprocessing of XFEL data in real time.

Phase-retrieval and orientation-finding algorithms would greatly benefit from clean data. In Section 3.1, we carried out a sanity check to show that intensities processed by our model do not cause distortions in real space. In Section 3.3, we showed that EMC reliably converges to a solution using fewer patterns when provided with denoised and demasked data. This result is particularly interesting for studying rare or short-lived conformational states where data can be scarce. While these findings are promising, these algorithms are notoriously susceptible to biases and distortions in diffraction intensities. We would therefore not recommend U-Net outputs as an input to these algorithms without further studies. Still, the algorithm might already be very useful to improve online hit finding and pattern classification, where missing data can severely limit the analysis. For example, filling in the missing data makes it possible to calculate an autocorrelation directly, free from the strong artefacts caused by missing data and low signals. This result can also be used during phase retrieval, where the autocorrelation function is often used as an initial real-space constraint.

Data processing speed is crucial to X-ray single-particle imaging experiments where big data sets need to be processed online at high rates. Our U-Net can process images at 200 Hz on a modern GPU (RTX 2080 Ti). This rate is comparable to the current hit rates achieved during X-ray SPI experiments (Ayyer *et al.*, 2021 ▸).

This model proved to be particularly robust in handling diffraction patterns from nanoparticles that are not represented in size and shape within our data set, ensuring its application to restoring data from unknown structures. By tailoring our model to the restoration of diffraction intensities for specific protein classes (*e.g.* membrane proteins) with similar geometries, U-Net performance, while losing generality, could further improve on such a specific task. Ideally, a well trained network should handle different noise sources, background signals and masks. We believe our method could be quickly tuned to work under different experimental settings, as suggested by the short training time and its robustness to new data, providing an interesting tool for real-time data analysis during beamtimes. For future experimental applications where the background is significant, we think that model training should include dark runs to allow the network to learn from the specific background of the experiment and other sources of noise for data augmentation purposes.

## Supplementary Material

Supporting figures for data from geometrical shapes (cube, sphere, icosahedron) and circular beamstop. DOI: 10.1107/S1600576721012371/cw5034sup1.pdf

## Figures and Tables

**Table 1:** Average MSE and PSNR calculated with respect to simulated noiseless data The first column shows the intensity factor used when Poisson sampling normalized data. The second column is relative to the average metrics calculated for noisy patterns at the correspondent intensity factor. The last two columns report the average performance of the two methods.

	Average noisy pattern		Average U-Net		Average binary filter	
Intensity factor	MSE	PSNR	MSE	PSNR	MSE	PSNR
1000	3.8 × 10^−6^	54.2	8.5 × 10^−7^	60.7	3.5 × 10^−7^	64.6
500	7.6 × 10^−6^	51.2	1.4 × 10^−6^	58.5	6.8 × 10^−7^	61.7
100	3.7 × 10^−5^	44.3	3.0 × 10^−6^	55.2	2.8 × 10^−6^	55.5
50	7.4 × 10^−5^	41.3	5.9 × 10^−6^	52.3	5.0 × 10^−6^	53.0
10	3.8 × 10^−4^	34.2	1.2 × 10^−5^	49.2	2.1 × 10^−5^	46.7

**Table 2:** Mean squared errors for diffraction intensities of geometric shapes, at the lowest signal-to-noise ratio (intensity factor equal to 10)

Object	MSE	PSNR
Sphere	5.69 × 10^−6^	52.4
Cube	1.04 × 10^−5^	49.8
Icosahedron	7.34 × 10^−6^	51.3
PDB 1wcd (2.67 m)	1.71 × 10^−5^	47.7
PDB 1wcd (2.00 m)	5.10 × 10^−6^	52.9
PDB 3cji (2.67 m)	7.06 × 10^−6^	51.1
PDB 1p58 (2.67 m)	5.28 × 10^−6^	52.8
