# Supplementary material for: Noise reduction and mask removal neural network for X-ray single-particle imaging
Source: J Appl Crystallogr. 2022 Feb 1;55(Pt 1):122–32. doi: 10.1107/S1600576721012371 (PMC8805166; doi:10.1107/S1600576721012371)
Supplement: Supplementary file 1 [file j-55-00122-sup1.pdf]

JOURNAL OF APPLIED CRYSTALLOGRAPHY

Volume 55 (2022)

Supporting information for article:

Noise reduction and mask removal neural network for X-ray single-particle imaging

Alfredo Bellisario, Filipe R. N. C. Maia and Tomas Ekeberg

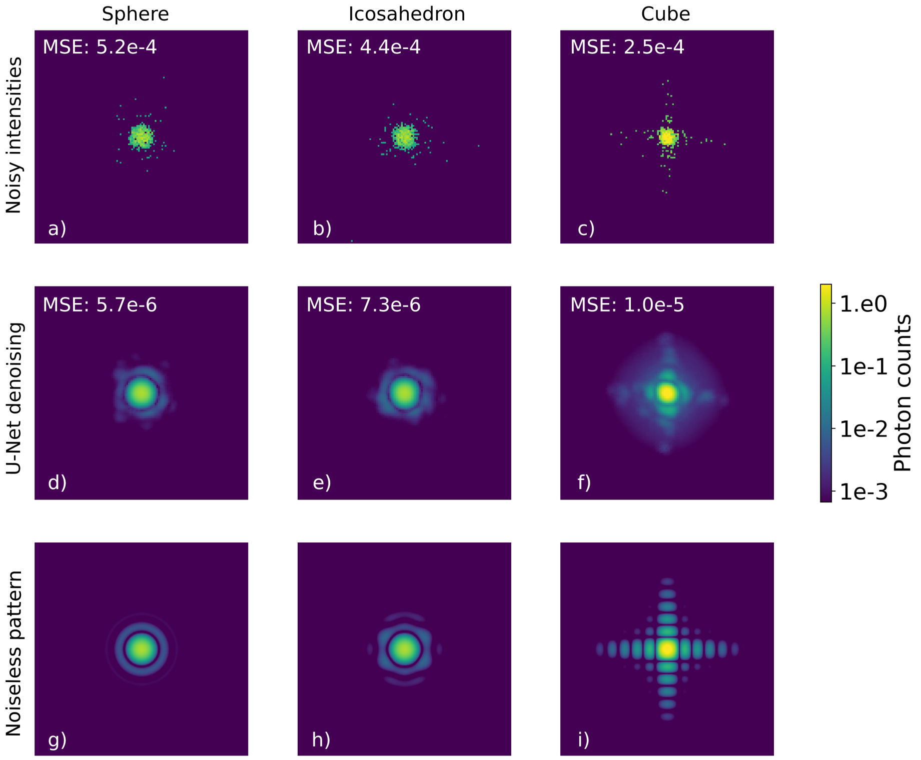

Figure S1: Diffraction patterns from geometric shapes, logarithmic scale colormap. (a-c) Diffraction patterns from respectively a sphere, an icosahedron, and a cube at intensity factor 10. (d-f) U-Net denoised intensities. (g-i) Noiseless diffraction patterns

.

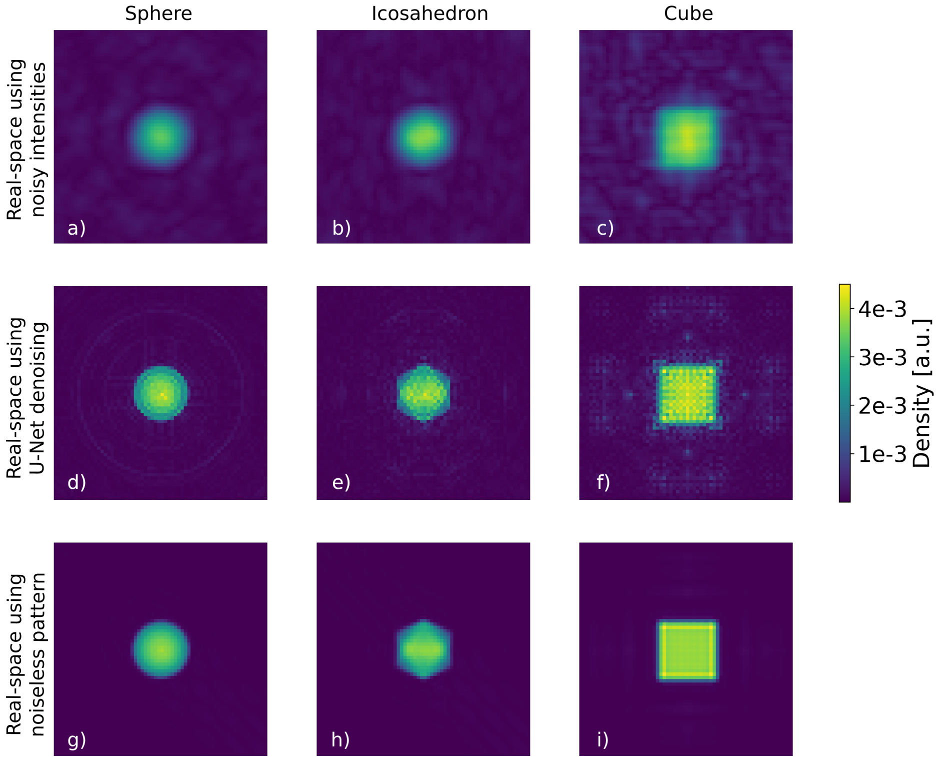

Figure S2: Real-space densities obtained assuming known phases. (a-c) Reconstructions obtained from noisy intensities. (d-f) Reconstructions obtained from intensities denoised using U-Net. (g-i) True densities.

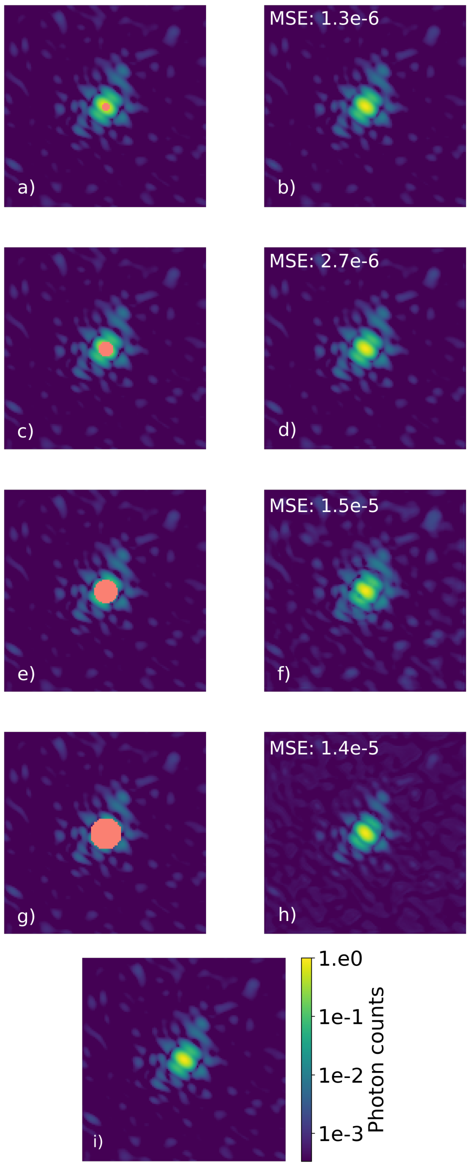

Figure S3: Diffraction patterns for PDB deposited protein structure 4TV4, logarithmic scale colormap. Color orange is used in the left column to highlight masked pixels. (a,c,e,g) simulated diffraction patterns with circular beamstop respectively 5, 10, 15, and 20 pixels wide. (b,d,f,g) U-Net demasked output. (i) simulated diffraction intensities, without mask.

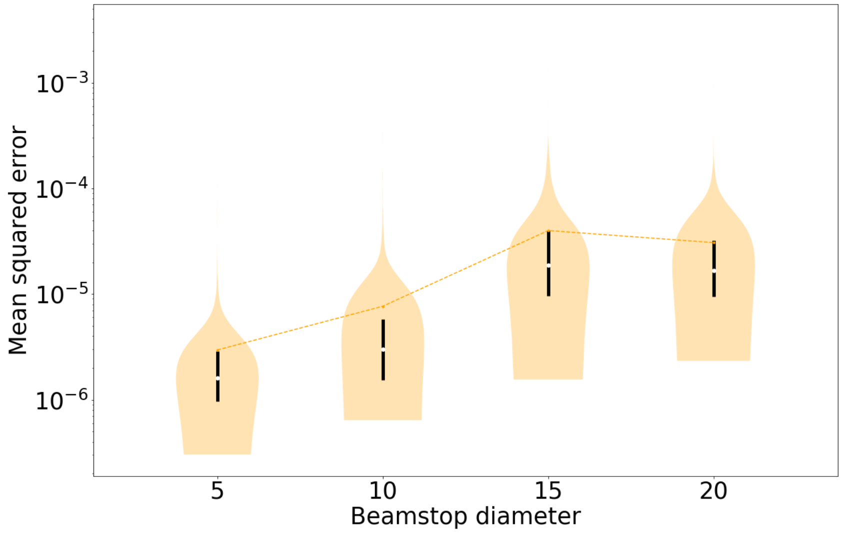

Figure S4: Violin plot of the MSE calculated over all of the 1000 patterns in the test dataset for increasing mask diameter.
